# False Memories in Native and Foreign Languages

**DOI:** 10.3389/fpsyg.2021.716336

**Published:** 2021-09-28

**Authors:** Aleksandra Dolgoarshinnaia, Beatriz Martin-Luengo

**Affiliations:** Centre for Cognition and Decision Making, Institute for Cognitive Neuroscience, HSE University, Moscow, Russia

**Keywords:** eyewitness memory, false memory, bilingualism, misinformation effect, source monitoring

## Abstract

Human memory is prone to memory errors and distortion. Evidence from studies on cognitive functions in bilinguals indicates that they might be prone to different types of memory errors compared to monolinguals; however, the effect of language in false memories is still understudied. Source monitoring processes required for proper memory functioning, presumably, rely on inhibitory control, which is also heavily utilized by bilinguals. Moreover, it is suggested that thinking in a second language leads to more systematic and deliberate reasoning. All these results lead to expect that bilinguals are more analytical when processing information in their second language overcoming some memory errors depending on the language of information. To test this hypothesis, we run a classical misinformation experiment with an explicit source monitoring task with a sample of Russian–English bilinguals. The language of the misinformation presentation did not affect the degree of the misinformation effect between the Russian and English languages. Source monitoring demonstrated an overall higher accuracy for attributions to the English source over the Russian source. Furthermore, analysis on incorrect source attributions showed that when participants misattributed the sources of false information (English or Russian narrative), they favored the Russian source over the not presented condition. Taken together, these results imply that high proficiency in the second language does not affect misinformation and that information processing and memory monitoring in bilinguals can differ depending on the language of the information, which seems to lead to some memory errors and not others.

## Introduction

Globalization and worldwide migration lead to an increase in the percentage of the population that simultaneously speaks two and more languages. Evidence from studies on cognitive functions in bilinguals indicates that they might be prone to different types of memory errors compared to monolinguals (Marian and Neisser, 2000; Boroditsky et al., 2003, 2009, 2019; Fausey and Boroditsky, 2011). These individuals may face a situation where they have to testify in a foreign country using their second language to communicate. Unlike monolingual witnesses, they can be exposed to misleading information from sources in several languages. Results of the existing studies are not enough to lead to any sort of conclusion on how such situations might affect their memories and recollections. Moreover, the misinformation effect is rarely checked through explicit source monitoring tasks, as it is usually implied that if the misinformation effect is present, source confusion has occurred. Furthermore, to our knowledge, only few studies investigated possible implications in bilingual populations (Tosun et al., 2013; Ünal et al., 2016). However, these studies investigated the effects of grammatically expressed evidentiality of the action agent. While undoubtedly important, this research is specific to particular languages with such expressed features (e.g., Turkish), and the effect in other languages remains unclear. Therefore, the present research aimed to investigate the effect the language of the presentation can have on memory errors, such as the misinformation effect and source misattributions.

The misinformation paradigm is one of the available research designs that enable the research on false memories and misinformation effect (Loftus and Palmer, 1974; Loftus, 1975; Loftus and Guido, 1975; Loftus and Hoffman, 1989). In short, the misinformation effect occurs when postevent false information is wrongly reported as the original. In the laboratory, the misinformation paradigm is operationalized as follows: witnessing the original event (Phase 1), exposure to false information about the event (Phase 2), and memory test to measure the acceptance of false information (Phase 3). As a result, participants report that they remember false information from witnessing the event rather than the later sources. The degree of memory distortion and the resulting false memories can vary due to a variety of factors. Susceptibility to misinformation was found to increase with age (Ceci et al., 1987; Loftus, 2005) and the time passed between witnessing the event, exposure to false information, and recollection of the event (Loftus, 2005). On the other hand, misinformation acceptance can decrease if people are warned that they have received or will receive information that is not entirely correct (Echterhoff et al., 2005; Loftus, 2005). Finally, the nature of the details is also important; schema-consistent false information is easier accepted than schema-inconsistent or schema irrelevant information (Tuckey and Brewer, 2003); furthermore, memory for the central information is generally better remembered than for the peripheral (Luna and Migueles, 2009); however, it can also differ depending on the emotional arousal and valence of the situation and information (Christianson and Loftus, 1991; Porter et al., 2003).

One of the most accepted explanations for the misinformation effect is “source monitoring errors,” that is, errors in the attribution of the information (Lindsay et al., 1991; Johnson et al., 1993; Johnson, 1997; Luna and Martín-Luengo, 2013). There are two key mechanisms underlying the failures in source discrimination. First, depending on the quality of the available information (perceptual cues and schema consistency or inconsistency), the credibility of the memory is either defined through rapid and heuristic processing or requires more deliberate and systematic processing (Johnson et al., 1993). It is considered that source attributions are mainly made heuristically (“System-1” processing), whereas systematic reasoning (“System-2” reasoning) is engaged to a lesser degree, but both can be activated for “more careful” judgments. The decision criteria that are used in both, the System-1 and System-2 processes differ, and the criterion for the latter is stricter. Thus, when both the heuristic and systematic processes are used, the criterion tightens, and better judgments are made. Generally, the criteria are rather flexible, can be influenced by biases, meta-memory assumptions, and vary across different types of events and situations (Johnson et al., 1993). Second, research on suggestibility and reality monitoring in children revealed that inhibitory control can be another important mechanism underlying the source monitoring processes. Several studies showed that younger children experience difficulties when performing source monitoring tasks, and the performance usually improves with age (Foley et al., 1983). Neuroscientific findings associate source monitoring processes to activations in the prefrontal cortex (Johnson et al., 1993; Ruffman et al., 2001), which is associated with executive functioning including the inhibitory control (Waltz et al., 1999). Developmentally, the prefrontal cortex matures later compared to the other regions of the brain (Diamond, 2002), and its maturation is correlated with the ability of the children to inhibit task-irrelevant or competing for information (Sinopoli and Dennis, 2012). Moreover, several studies investigated the relationship between these processes directly by testing the source memory performance and measuring the level of inhibitory control (Ruffman et al., 2001). The results of these studies indicate that greater inhibitory control is positively correlated with better performance on source monitoring tasks.

The effect of language on memory, specifically, episodic memory was repeatedly observed within and between the languages. Many studies investigated the effect of wording in leading questions similar to those asked witnesses during interrogations (Loftus and Palmer, 1974; Loftus, 1975; Loftus and Guido, 1975). These studies demonstrated that even slight lexical variations in questions, such as the usage of synonyms can remarkably influence the answer of an individual and the recollection of an event. Also, the linguistic influence on the memory of an individual remains persistent across different language groups as shown by further studies. For example, Fausey and Boroditsky, 2011 compared English and Spanish as well as English and Japanese monolinguals in their recall of intentional and accidental events and the agent who acted in these events. Observed cross-linguistic differences in memory performance were described as caused by the different grammar patterns specific to these languages, e.g., more frequent use of an agent in the English language compared to Japanese or Spanish. Similar studies also explored the effect in several other languages, including Turkish (Aydin and Ceci, 2013) and Indonesian (Fausey and Boroditsky, 2011), as well as different linguistic characteristics, such as the usage of definite or indefinite articles (Loftus, 1975), grammar tenses (Boroditsky et al., 2019), or gender (Boroditsky et al., 2003), all showing variations in the memory performance attributed to the specific linguistic characteristics.

These findings and a general rise of interest in the cognitive functions in bi and multilingual populations sparked the interest in memory processing and memory mistakes in individuals who utilize more than one language. Providing a mental representation for the event, language can be labeled as a contextual cue which affects the way information is encoded and retrieved, implying that in bilinguals, the memory of an event can be better when encoded and retrieved in the same language as opposed to situations when languages are inconsistent (Schroeder and Marian, 2014). Furthermore, it has been long argued that linguistic characteristics, being specific to a particular language, can shape the thoughts and behavior of an individual (Schroeder and Marian, 2014). As per the thinking-for-speaking concept proposed by Slobin, when speaking, a person can direct his attention to particular details through syntactic structures that are established by the language he speaks (Slobin, 1987). This implies that the structure of the language and the cultural representations associated with this language can create a certain perspective through which a person processes information. When bilinguals are concerned, it also means that encoding can be different depending on the language they use at the moment. Yet, while episodic and autobiographical memory in bilinguals has been widely researched and differences in encoding and retrieval processes were observed (Boroditsky et al., 2009; Fausey and Boroditsky, 2011; Aydin and Ceci, 2013), there have been only a handful of studies investigating the suggestibility in bilinguals (Shaw et al., 1997; Smith et al., 2017; Calvillo and Mills, 2020). Although misinformation effect was present in all these cross-linguistic studies, the results regarding the influence of the language on the endorsement of false information show inconsistent findings, reporting no significant differences between the conditions (Shaw et al., 1997) or explaining the results in relation to the different levels of proficiency between the languages (Calvillo and Mills, 2020). At the same time, the potential effects of proficiency levels of the second language were not yet explored. All of this, therefore, demonstrates the need for further research of the phenomenon.

Research on source monitoring has also advanced in the last decade. Nevertheless, it is still rarely explicitly tested in the misinformation paradigm, as the presence of the effect implies that source confusion has occurred. Moreover, it is still unclear whether source monitoring processes can be affected by language and bilingualism. Several studies (Tosun et al., 2013; Ünal et al., 2016) investigated the effects of grammatically expressed agency; however, as this grammar feature is present in some languages and not others, the results cannot be generalized. Thus, language influence on source monitoring processes requires further examination.

In sum, research on the effects of bilingualism on cognitive functions, such as decision-making, attention, and memory processing suggests that bilinguals may have certain advantages in performing non-linguistic tasks due to their more trained inhibitory control because of the selection of languages compared to monolinguals (Bialystok et al., 2005; Weissberger et al., 2015). Furthermore, bilinguals could rely on more analytical and deliberate System-2 processing as opposed to heuristic System-1 (Caldwell-Harris, 2014; Costa et al., 2014; Hayakawa et al., 2016, 2017; Bialek et al., 2019). Therefore, we suggested that bilinguals would be less susceptible to misleading details in their second language, as their monitoring mechanisms would be more engaged in information processing. Thus, the misinformation effect would be reduced. Following this hypothesis, we expected a lower level of accuracy on the recognition task in the native language compared to the foreign language. Alternatively, we also hypothesized that bilinguals could be more susceptible to misleading information in their second language, as their cognitive resources would be more engaged with the linguistic processing of the information, therefore leaving fewer resources for monitoring the source of this information. In this case, we could expect a lower level of accuracy on the recognition task in foreign, rather than in the native language. Our second expectation was that due to a higher degree of misinformation effect in the native language, a higher confidence rate for incorrect answers on the recognition task for the native language would be observed. More expectations were made regarding the source-monitoring judgments. Based on the previous expectations of observable misinformation effect, we expected to see lower accuracy for misleading items in the native language compared to foreign for the source monitoring task, as the presence of the misinformation effect implies that memory for misleading information is treated as a memory for the original event. In relation to that, we expected higher confidence ratings for these items on the source monitoring task. In the case of the alternative hypothesis, the pattern should be reversed with lower accuracy for misleading items in a foreign language. To test these hypotheses, we run an experiment using the misinformation paradigm with the classical three phases: presentation of the original information (no sound video), exposure to misleading information (narratives describing the event of the video in different languages), and memory test in this case with an explicit source monitoring task.

## Materials and Methods

### Participants

A total of 70 bilingual volunteers (46 women, mean age = 24.2, *SD* = 4.63) recruited through social media took part in this online study, created, and hosted in the Gorilla Experiment Builder (Anwyl-Irvine et al., 2020). Participants received a small monetary reward (250 rubles) for the successful completion of the experiment. *A priori* analysis conducted in GPower (version 3.1) determined the sample size as 48 participants (effect size = 0.40, power = 0.80, and alpha = 0.05). The selection requirements for the participants included having Russian as the native language, speaking intermediate or higher level of English proficiency (according to the Common European Framework of Reference, CEFR), having no prior experience of immersion in an entirely English-speaking environment for a prolonged period of time (more than 5 months) (Costa et al., 2014). All the participants completed an online Cambridge Assessment test (www.cambridgeenglish.org), and nine participants who scored below 16 points (Pre-Intermediate and lower, CEFR) on the Cambridge test were excluded, leaving mainly high-proficient participants (mean proficiency = 20 out of 25 points, *SD* = 2.9). Five more were excluded due to the violation of the English-speaking environment requirement. The final sample reported in this study consisted of 56 participants (40 women, mean age = 24.1, *SD* = 4.66).

### Normative Study and Materials

**Figure 1:**
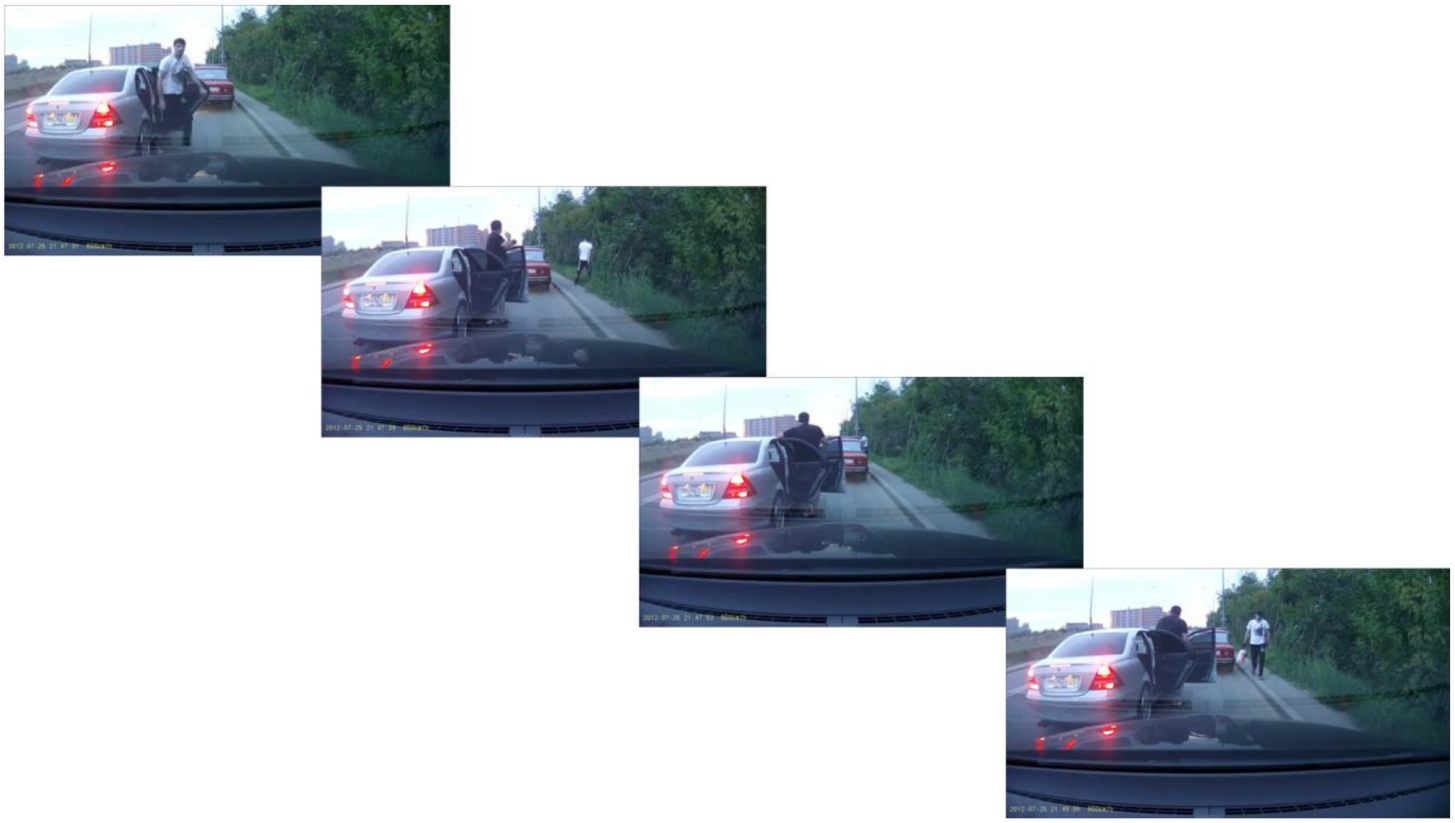
The temporal sequence of footage of a car robbery. The video is recorded from a camera in a car parked at the side of the road. It captures a gray car parked in front of the car with a camera. A young man wearing a white T-shirt leaves the gray car and enters the bushes on the side of the road. At the same time, a second man leaves the car but stays beside it looking around. The young man then reappears from the bushes hiding something behind his back and heads toward one of the cars parked in front. After a while, he returns carrying several plastic bags. Both men then enter the gray car and drive away.

As the main stimulus, we used 61-s real footage of a car robbery (as shown in Figure 1 for the video outline). First, true items were extracted directly from the contents of the video (11). Then, false items (20) were created that either distorted the information of the video (for example, white T-shirt instead of black) or did not appear in the video but were plausible to happen in that environment.

Before the main experiment, we ran a normative study to guarantee equal memorability to both true and false items. The normative study was conducted in Russian, with 20 volunteers (15 women, mean age = 25, and *SD* = 3.34), who did not take part in the main experiment. Participants of the normative study were not required to watch the video; instead, they read two narratives describing the event in the video. Each narrative contained 10 critical details, which were considered true for the normative study and were going to be used as misleading in the main experiment. To check the memory of these details, 11 false control items were used on the recognition task. These false items were originally identified as critical true elements in the video for the main experiment. The narratives were presented separately for the two groups of participants, one narrative for one group (each group consisting of 10 participants). This separation in two groups is based on the counterbalance of the elements in the design of the main experiment as they should not have referred to the same object, person, or modality, and should not have interfered with each other in one set of the narratives. Procedurally, the participants were required to read a narrative, and after a distractor task to perform a recognition test, they had to indicate whether a particular piece of information was present in the text or not (True or False) and rate their confidence in their answer on a 1 (not confident at all) to 10 (totally confident) scale. The normative study confirmed a similar memorability of both true and false items (*p* > 0.05), which allowed us to use all 32 previously identified items in the further experiment.

For the main experiment, a total of 31 details were used (Supplementary Appendix A). The true items (11) were the same for all the conditions. Counterbalancing false items (20), some of them were used as misleading information introduced during the misinformation stage and some were used as false control items on the recognition task. For introducing misleading information, we created four pairs of narratives in Russian and English to counterbalance the misleading and control details as well as the language in which they were presented. Each narrative contained five misleading details (making it 10 for a set).

### Procedure

**Figure 2:**
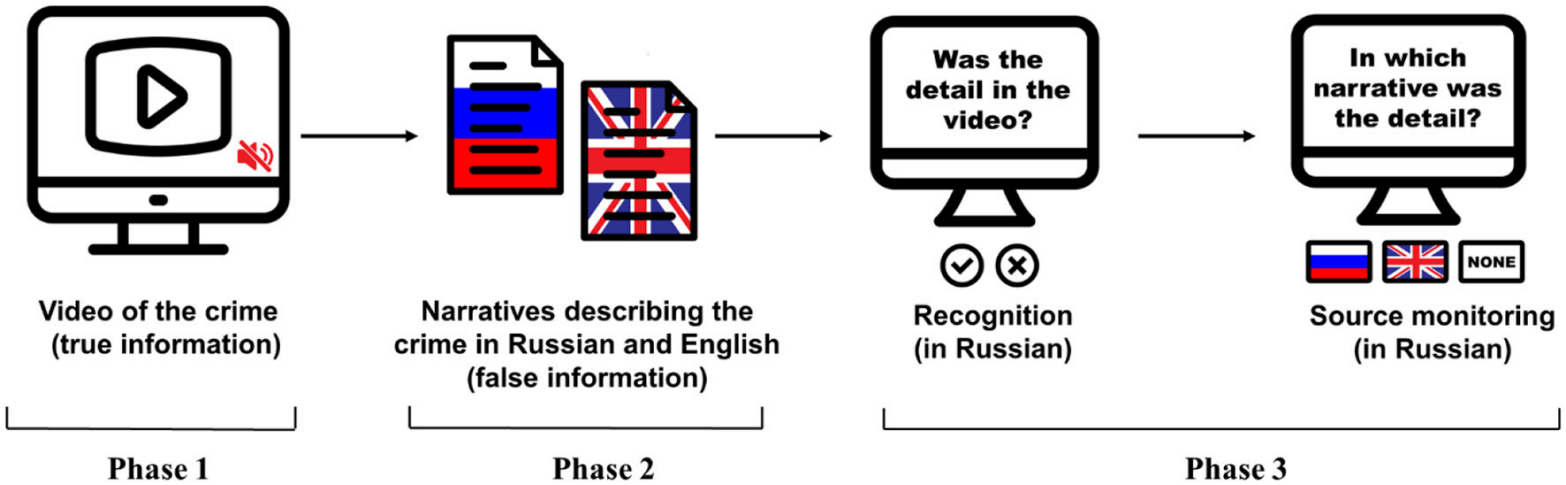
Experimental design. Phase 1: encoding of true information from a video. Phase 2: introduction of misinformation from two narratives (Russian and English). Phase 3: true/false recognition + source monitoring (Russian narrative, English narrative, none).

Participants accessed the experiment *via* a link shared by the experimenter. They read and explicitly stated their agreement with an electronic consent form before starting the experiment. Then they filled in a social and linguistic questionnaire adopted from Marian et al. (2007). After that, the participants were randomly assigned to one of the four counterbalanced conditions and completed a misinformation paradigm consisting of the following stages: witnessing the event, encountering misleading information, recognition test, and explicit source monitoring test (Figure 2).

First, the participants watched the 1-min long video of a car robbery. Immediately after it, they were asked to perform a series of distracting numeric tasks that took ~4 min to complete. Upon completing these numeric tasks, the participants read a pair of narrative accounts of what took place in the video. The narratives were described to the participants as reports made by two witnesses of the crime, a Russian native speaker and his friend who happened to be an English native, reported in the corresponding languages. Each of the narratives consisted of five misleading details about the event. Then the participants performed another series of distracting numeric tasks analogous to the first one and of the same duration. Next, the participants completed a recognition test along with the source-monitoring test. For the recognition test, they indicated whether the detail or item described in a statement was present in the original video (true or false) and indicated the level of confidence in their answer on a scale from 1 (not confident at all) to 10 (totally confident). Immediately after the recognition question, the participants performed a source monitoring task for which they had to indicate whether the detail or item from the previous question was mentioned in the Russian or English narrative, or it did not appear in any of the narratives (for true and false control items). Participants rated their confidence for their answers on this question again from 1 (not confident at all) to 10 (totally confident). Finally, the participants had to complete a general English proficiency test adapted from an online Cambridge Assessment.

### Statistical Analysis

#### Misinformation

Typically, the misinformation effect is checked and calculated as a difference in the proportion of false alarm rates of misleading and control items reflecting the acceptance of misinformation. Due to the specificity of design and materials, in particular, the absence of control items that would fully match the conditions (i.e., there were no control English items), a 2 × 2 ANOVA was not feasible. Instead, we made direct comparisons on the proportions of false alarms for misleading and control items by *t*-tests.

#### Source Monitoring

To analyze the performances on source monitoring task and corresponding confidence ratings, we ran a 3 × 3 repeated measures ANOVA with correct and incorrect source attributions of the participants (Russian narrative, English narrative, and None, where information was not presented in the narratives). We then investigated the differences within these factors by running a repeated measures ANOVA for correct source attributions (Russian-to-Russian, English-to-English, and None-to-None) and Student's *t*-tests for incorrect source attributions (Russian-to-English, Russian-to-None, English-to-Russian, English-to-None, None-to-Russian, and None-to-English).

## Results

Below are reported analyses of ANOVA on the recognition and source monitoring tasks and their corresponding confidence ratings. When appropriate two-tailed pairwise comparisons with the Student's *t*-test were used. For pairwise comparisons, we applied Bonferroni correction to avoid Type I error for conducting multiple statistical tests. Partial-eta squared (η^2^p) and Cohen's d (*d*) are also reported as the measures of effect size.

### Misinformation

First, the misinformation effect was confirmed since the proportion of false alarms for misleading information (*M* = 0.32, *SD* = 0.19) were significantly higher than for the control items (*M* = 0.21, *SD* = 0.12), *t*_(55)_ = 5.134, *p* < 0.001, and *d* = 0.686. Moreover, mean confidence ratings were higher for misleading (*M* = 24.34, *SD* = 16.70), than for the control items (*M* = 14.77, *SD* = 10.37), *t*_(55)_ = 4.392, *p* < 0.001, and *d* = 0.587. However, misinformation effect did not differ between the Russian (*M* = 0.13, *SD* = 0.11) and English languages (*M* = 0.13, *SD* = 0.11), *t*_(55)_ = 1.169, *p* = 0.866, and *d* = 0.023.

### Source Monitoring

**Figure 3:**
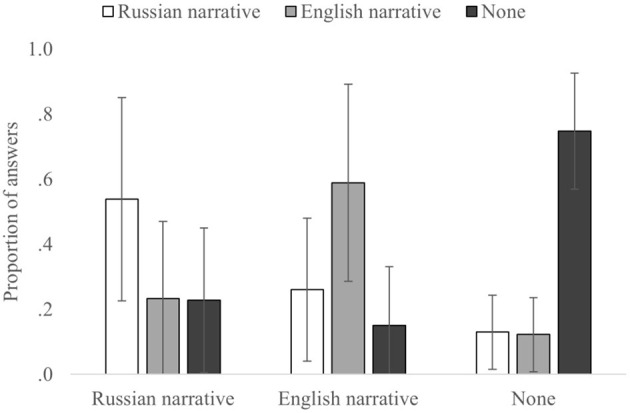
The proportion of answers for each correct and incorrect source. Error bars indicate SD.

First, a 3 × 3 ANOVA on the correct and incorrect source attributions of the participants (Table 1, Figure 3) showed no main effect for the correct source, *F*_(2,110)_ = 0.003, *p* = 0.999, and η^2^_p_= 0.00006; however, it showed main effect for the incorrect source, *F*_(2,110)_ = 3.910, *p* = 0.023, and η^2^_p_= 0.066, and their interaction, *F*_(4,220)_ = 73.580, *p* < 0.0001, and η^2^_p_= 0.572.

**Table 1:** Means (SD) of correct and incorrect source attributions.

**Actual source**	**Participants' attributions**		
	**Russian narrative**	**English narrative**	**None**
Russian narrative	0.54 (0.31)	0.23 (0.23)	0.23 (0.22)
English narrative	0.26 (0.21)	0.59 (0.30)	0.15 (0.18)
None	0.13 (0.11)	0.12 (0.10)	0.75 (0.18)

For the correct source attributions (diagonal in Table 1), we found significant differences, *F*_(2,110)_ = 12.250, *p* < 0.0001, and η^2^_p_= 0.183, None-to-None attributions were significantly higher than Russian-to-Russian [*t*_(55)_ = −5.733, *p* < 0.001, and *d* = −0.766] and English-to-English [*t*_(55)_ = −3.890, *p* < 0.001, and *d* = −0.520]. No significant differences were found in correct attributions between Russian-to-Russian and English-to-English [*t*_(55)_ = −0.958, *p* = 0.342, and *d* = −0.128].

Analysis on incorrect attributions for the Russian source (when Russian narrative was the correct source), showed that the differences between Russian-to-English [*t*_(55)_ =4.493, *p* < 0.001, and *d* = 0.600] and Russian-to-None [*t*_(55)_ = 4.766, *p* < 0.001, and *d* = 0.637] source misattributions were not significant [*t*_(55)_ = 0.131, *p* = 0.896, and *d* = 0.018]. Similarly, for the None source (when information was not presented in the narratives), differences between None-to-Russian [*t*_(55)_ = 16.403, *p* < 0.001, and *d* = 2.192] and None-to-English [*t*_(55)_ = 17.522, *p* < 0.001, and *d* = 2.341] source misattributions were also insignificant [*t*_(55)_ = 0.442 *p* = 0.639, and *d* = 0.063]. On the other hand, for the English source (when the English narrative was the correct source), the proportion of English-to-Russian [*t*_(55)_ = 4.939, *p* < 0.001, and *d* = 0.660] misattributions was higher than that of the English-to-None [*t*_(55)_ = 7.323, *p* < 0.001, and *d* = 0.979] misattributions [*t*_(55)_ = 3.129, *p* = 0.003, and *d* = 0.418].

To summarize, these results revealed several patterns for source attributions in the misinformation paradigm. For correct source attributions, they were significantly higher than incorrect source attributions in all the conditions; however, there was no significant difference between the Russian and English sources. Further analyses on incorrect attributions showed no significant results for the Russian and None sources, but not for the English source misattributions to the Russian source, which were significantly higher than the English source misattributions to the None source.

### Source Monitoring Confidence

**Figure 4:**
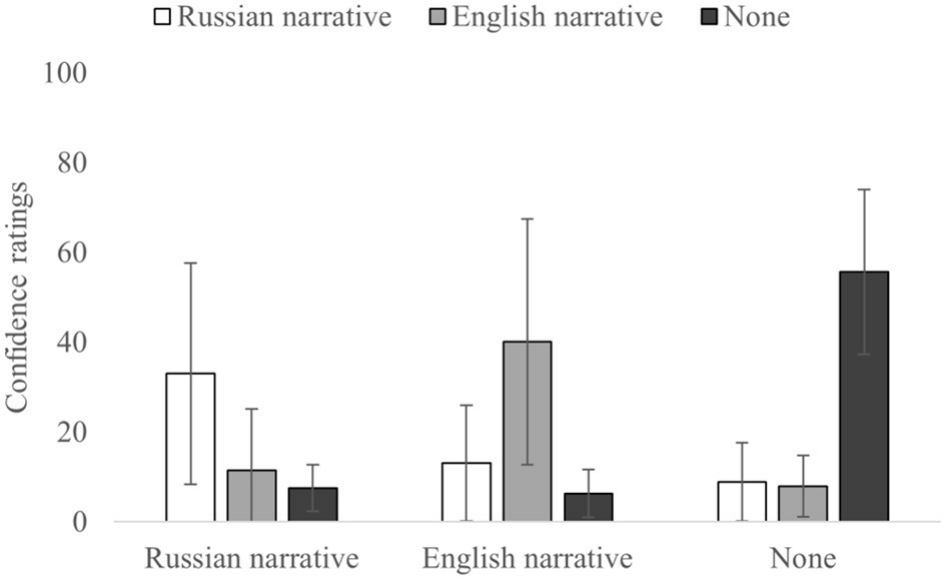
Confidence ratings for each correct and incorrect source. Error bars indicate SD.

First, a 3 × 3 ANOVA for confidence ratings of correctly and incorrectly attributed sources (Table 2, Figure 4) showed significant main effect on confidence when the source was identified correctly, *F*_(2,110)_ = 18.501, *p* < 0.001, and η^2^_p_= 0.252, or incorrectly, *F*_(2,110)_ = 4.357, *p* = 0.015, and η^2^_p_=0.073, as well as their interaction, *F*_(4,220)_ = 100.599, *p* < 0.00001, and η^2^_p_ = 0.647.

**Table 2:** Means (SD) of confidence ratings for correct and incorrect source attributions.

**Actual source**	**Participants' attributions**		
	**Russian narrative**	**English narrative**	**None**
Russian narrative	33.0 (24.61)	11.39 (13.69)	7.54 (5.21)
English narrative	13.04 (12.88)	40.07 (27.38)	6.28 (5.33)
None	8.86 (8.74)	7.93 (6.81)	55.65 (18.36)

For the correct source attributions (diagonal in Table 2), there were differences, *F*_(2,110)_ = 17.569, *p* < 0.0001, and η^2^_p_= 0.242. In particular, the confidence for None-to-None attributions was higher than for Russian-to-Russian [*t*_(55)_ = −7.109, *p* < 0.001, and *d* = −0.950] and English-to-English [*t*_(55)_ = −4.013, *p* < 0.001, and *d* = −0.536]. At the same time, there was no difference in confidence ratings for correct source attributions between Russian-to-Russian and English-to-English [*t*_(55)_ = −1.557, *p* = 0.125, and *d* = −0.208].

Analysis on confidence ratings for the incorrect attributions for the Russian source (when the Russian narrative was the correct source) demonstrated that differences in confidence between Russian-to-English [*t*_(55)_ = 5.058, *p* < 0.001, and *d* =0.676] and Russian-to-None [*t*_(55)_ = 6.716, *p* < 0.001, and *d* = 0.897] source misattributions were not significant [*t*_(55)_ = 1.867, *p* = 0.067, *d* = 0.250]. Similarly, for the None source (when information was not presented in the narratives), differences in confidence between None-to-Russian [*t*_(55)_ = 14.494, *p* < 0.001, and *d* = 1.937] and None-to-English [*t*_(55)_ = 15.932, *p* < 0.001, and *d* = 2.129] source misattributions were also not significant [*t*_(55)_ = 0.760, *p* = 0.451, and *d* = 0.102]. Finally, for the English source (when English narrative was the correct source), confidence ratings were higher for the English-to-Russian [*t*_(55)_ = 5.974, *p* < 0.001, and *d* = 0.798] and the English-to-None [*t*_(55)_ = 8.088, *p* < 0.001, and *d* = 1.081] misattributions, [*t*_(55)_ = 3.540, *p* < 0.001, and *d* = 0.473].

To sum up, the results of the analyses on confidence complemented the results of source monitoring analyses and showed similar patterns. First, confidence for correct source attributions was higher than that for incorrect source attributions for all the conditions. Second, there was no significant difference in the confidence between correct attributions for the Russian and English sources. Finally, analyses on the confidence for misattributions showed no significant differences for the Russian and None sources; however, for the English source, the confidence was higher when incorrect attributions were made in favor of the Russian source rather than the None source, which fully mirrors the results of the source monitoring analyses.

### Additional Analysis by the Level of Proficiency

One of the main criteria for the participants in this study was intermediate or higher levels of English language proficiency, which was necessary to ensure that the participants would not experience any difficulties in understanding the materials. Nevertheless, in an attempt to further explore how different levels of language proficiency can affect memory performance for misinformation and source monitoring, we ran an additional analysis between the groups of participants with intermediate (scoring from 16 to 19) and higher levels of proficiency (scoring from 20 to 25). Each group consisted of 28 participants. Mean proficiency for the Intermediate group was 17.39 (*SD* = 1.1) out of 25 points, and for the High proficient group, it was 22.6 (*SD* = 1.5) points out of 25.

Analysis on the false alarm rates (as shown in Table 3), confirmed the acceptance of misinformation in both the groups [Int: *t*_(27)_ = 4.740, *p* < 0.001, and *d* = 0.896; High: *t*_(27)_ = 2.748, *p* = 0.011, and *d* = 0.519]. The degree of misinformation accepted from the Russian and English languages did not differ within the groups [Int: *t*_(27)_ = 1.622, *p* = 0.116, and *d* = 0.307; High: *t*_(27)_ = −1.451, *p* = 0.158, and *d* = −0.274]. However, comparison between the level of groups revealed that the degree of misinformation acceptance from the English language was significantly higher in the High proficient group compared to the Intermediate group, *t*_(27)_ = 2.460, *p* = 0.021, and *d* = 0. 465.

**Table 3:** Means (SD) of false alarm rates for control and misleading items for Intermediate and High proficient groups.

**Level of proficiency**	**FAs control**	**FAs misleading**		
		**Overall**	**Russian origin**	**English origin**
Intermediate	0.18 (0.12)	0.32 (0.21)	0.14 (0.12)	09 (0.11)
High proficient	0.23 (0.12)	0.33 (0.17)	0.13 (0.10)	0.17 (0.11)

**Figure 5:**
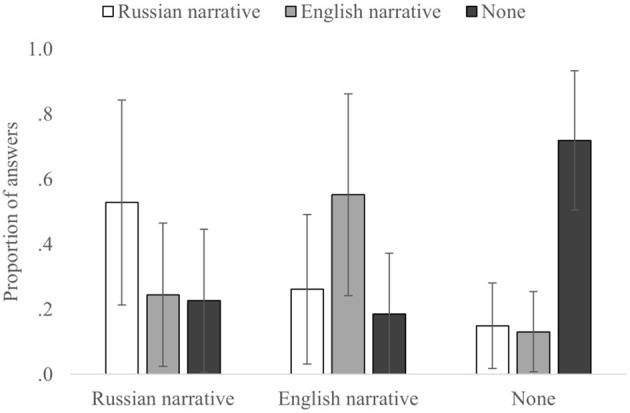
The proportion of answers for each correct and incorrect source for the Intermediate proficiency group. Error bars indicate SD.

**Figure 6:**
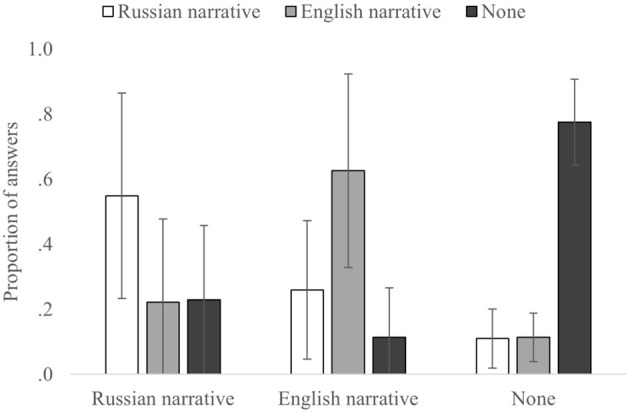
The proportion of answers for each correct and incorrect source for the High proficiency group. Error bars indicate SD.

Further analysis on the source monitoring within the proficiency groups demonstrated patterns similar to the results of the main analysis with the full sample (Table 4, Figures 5, 6). In both the groups, 3 × 3 ANOVA with the correct and incorrect source attributions of the participants showed no main effect for the correct source [Int: *F*_(2,54)_ = 2.681, *p* = 0.078, and η^2^_p_= 0.090; High: *F*_(2,54)_ = 0.458, *p* = 0.635, and η^2^_p_= 0.017] and incorrect source [Int: *F*_(2,54)_ = 1.940, *p* = 0.154, and η^2^_p_= 0.067; High: *F*_(2,54)_ = 1.999, *p* = 0.145, and η^2^_p_= 0.069]; at the same time, their interaction was significant [Int: *F*_(2,54)_ = 28.700, *p* < 0.0001, and η^2^_p_= 0.515; High: *F*_(2,54)_ = 46.088, *p* < 0.0001, and η^2^_p_= 0.631].

**Table 4:** Means (SD) of correct and incorrect source attributions for Intermediate (Int) and High proficiency groups.

**Actual source**	**Participants' attributions**					
	**Russian narrative**		**English narrative**		**None**	
	**Int**	**High**	**Int**	**High**	**Int**	**High**
Russian narrative	0.53 (0.31)	0.55 (0.32)	0.25 (0.22)	0.22 (0.25)	0.23 (0.22)	0.23 (0.23)
English narrative	0.26 (0.23)	0.26 (0.21)	0.55 (0.31)	0.63 (0.30)	0.19 (0.19)	0.11 (0.15)
None	0.15 (0.13)	0.11 (0.09)	0.13 (0.12)	0.11 (0.08)	0.72 (0.21)	0.78 (0.13)

For the correct source attributions (diagonal in Table 4), for both the groups, None-to-None attributions were significantly higher than the Russian-to-Russian [Int: *t*_(27)_ = −3.682, *p* = 0.001, and *d* = −0.501; High: *t*_(27)_ = −4.342, *p* < 0.001, and *d* = −0.821] and English-to-English attributions [Int: *t*_(27)_ = −2.649, *p* = 0.013, and *d* = −0.501; High: *t*_(27)_ = −2.827, *p* = 0.009, and *d* = −0.534]. No significant differences were found in correct attributions between the Russian-to-Russian and English-to-English [Int: *t*_(27)_ = −0.284, *p* =0.779, and *d* = −0.054; High: *t*_(27)_ = −1.176, *p* = 0.250, and *d* = −0.222].

As for the incorrect source attributions, both the groups mostly followed the same patterns revealed by the main analyses; however, some minor differences were also found. For misattributions for the Russian source (when Russian narrative was the correct source), there was no significant difference between the Russian-to-English [Int: *t*_(27)_ = 3.021, *p* = 0.005, and *d* = 0.571; High: *t*_(27)_ = 3.285, *p* = 0.003, and *d* = 0.621] and Russian-to-None [Int: *t*_(27)_ = 3.213, *p* = 0.003, and *d* = 0.607; High: *t*_(27)_ = 3.456, *p* = 0.002, and *d* = 0.653] source misattributions [Int: *t*_(27)_ = 0.306, *p* = 0.761, and *d* = 0.058; High: *t*_(27)_ = −0.113, *p* = 0.911, and *d* = −0.021]. Similarly, for the None source (when information was not presented in the narratives), the difference between None-to-Russian [Int: *t*_(27)_ = 9.050, *p* < 0.001, and *d* = 1.710; High: *t*_(27)_ = 16.526, *p* < 0.001, and *d* = 3.123] and None-to-English [Int: *t*_(27)_ = 9.613, *p* < 0.001, and *d* = 1.817; High: *t*_(27)_ = 18.023, *p* < 0.001, and *d* = 3.406] source misattributions was also not significant [Int: *t*_(27)_ = 0.683, *p* = 0.500, and *d* = 0.129; High: *t*_(27)_ = −0.208, *p* = 0.837, and *d* = −0.039]. However, for the English source (when English narrative was the correct source), English-to-Russian misattributions [Int: *t*_(27)_ = 3.026, *p* = 0.005, and *d* = 0.572; High: *t*_(27)_ = 4.521, *p* < 0.001, and *d* = 0.854] were significantly higher than English-to-None misattributions [Int: *t*_(27)_ = 5.128, *p* < 0.001, and *d* = 0.780; High: *t*_(27)_ = 6.993, *p* < 0.001, and *d* = 1.322] only in High proficient group [Int: *t*_(27)_ = 1.313, *p* = 0.200, and *d* = 0.248; High: *t*_(27)_ = 3.993, *p* < 0.001, and *d* = 0.755].

**Figure 7:**
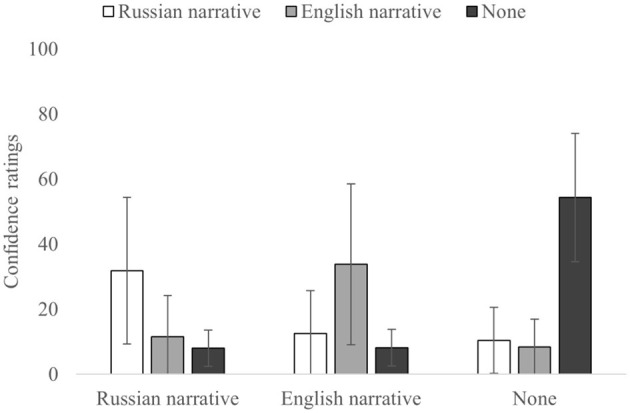
Confidence ratings for each correct and incorrect source for the Intermediate proficiency group. Error bars indicate SD.

**Figure 8:**
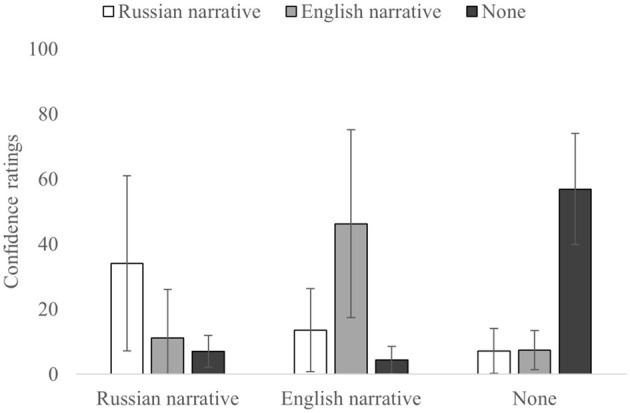
Confidence ratings for each correct and incorrect source for the High proficiency group. Error bars indicate SD.

Analysis on the confidence ratings of correctly and incorrectly attributed sources (Table 5; Figures 7, 8) showed the main effect for the correct source [Int: *F*_(2,54)_ = 13.655, *p* < 0.001, and η^2^_p_ = 0.336; High: *F*_(2,54)_ = 7.280, *p* = 0.002, and η^2^_p_= 00.212]. However, only the Intermediate group demonstrated the main effect for the incorrect source [Int: *F*_(2,54)_ = 3.964, *p* =0.025, and η^2^_p_= 0.128; High: *F*_(2,54)_ = 1.673, *p* = 0.197, and η^2^_p_= 0.058]. Meanwhile, the interaction was shown to be significant for both the groups [Int: *F*_(2,54)_ = 42.146, *p* < 0.0001, and η^2^_p_= 0.610; High: *F*_(2,54)_ = 59.860, *p* < 0.0001, and η^2^_p_= 0.689].

**Table 5:** Means (SD) of confidence ratings for correct and incorrect source attributions for Intermediate (Int) and High proficiency groups.

**Actual source**	**Participants' attributions**					
	**Russian narrative**		**English narrative**		**None**	
	**Int**	**High**	**Int**	**High**	**Int**	**High**
Russian narrative	31.86 (22.50)	34.14 (26.90)	11.57 (12.64)	11.21 (14.90)	8.04 (5.55)	7.04 (4.91)
English narrative	12.50 (13.25)	13.57 (12.72)	33.86 (24.73)	46.29 (28.89)	8.23 (5.66)	4.38 (4.24)
None	10.47 (10.17)	7.19 (6.83)	8.47 (7.61)	7.4 (6.0)	54.35 (19.75)	56.94 (17.11)

For the correct source attributions (diagonal in Table 5), for both the groups, confidence in None-to-None attributions was significantly higher than the Russian-to-Russian [Int: *t*_(27)_ = −6.097, *p* < 0.001, and *d* = −1.152; High: *t*_(27)_ = −4.329, *p* < 0.001, and *d* = −0.818]; however, confidence in None-to-None attributions was higher than for the English-to-English only in the Intermediate group [Int: *t*_(27)_ = −3.891, *p* < 0.001, and *d* = −0.735; High: *t*_(27)_ = −1.889, *p* = 0.070, and *d* = −0.357]. No significant differences were found for confidence in correct attributions between the Russian-to-Russian and English-to-English [Int: *t*_(27)_ = −0.325, *p* = 0.748, and *d* = −0.061; High: *t*_(27)_ = −1.826, *p* = 0.079, and *d* = −0.345].

Confidence for misattributions complemented previous results within the proficiency groups and mostly followed the patterns of the main analysis on confidence. For the Russian source (when Russian narrative was the correct source), in both the groups differences in confidence between Russian-to-English [Int: *t*_(27)_ = 3.857, *p* < 0.001, and *d* = 0.732; High: *t*_(27)_ = 3.351, *p* = 0.002, and *d* = 0.633] and Russian-to-None [Int: *t*_(27)_ = 4.741, *p* < 0.001, and *d* = 0.896; High: *t*_(27)_ = 4.710, *p* < 0.001, and *d* = 0.890] source misattributions were not significant [Int: *t*_(27)_ = 1.238, *p* = 0.226, and *d* = 0.234; High: *t*_(27)_ = 1.376, *p* = 0.180, and *d* = 0.260]. Similarly, for the None source (when information was not presented in the narratives), difference in the confidence ratings between None-to-Russian [Int: *t*_(27)_ = 8.728, *p* < 0.001, and *d* = 1.649; High: *t*_(27)_ = 12.216, *p* < 0.001, and *d* = 2.309] and None-to-English [Int: *t*_(27)_ = 9.527, *p* < 0.001, and *d* = 1.800; High: *t*_(27)_ = 13.694, *p* < 0.001, and *d* = 2.588] source misattributions were also not significant for both the groups [Int: *t*_(27)_ = 1.089, *p* = 0.286, and *d* = 0.206; High: *t*_(27)_ = −0.136, *p* = 0.893, and *d* = −0.026]. However, for the English source (when the English narrative was the correct source), confidence for English-to-Russian [Int: *t*_(27)_ = 3.488, *p* = 0.002, and *d* = 0.659; High: *t*_(27)_ = 4.956, *p* < 0.001, and *d* = 0.937] misattributions was significantly higher than for the English-to-None [Int: *t*_(27)_ = 4.761, *p* < 0.001, and *d* = 0.900; High: *t*_(27)_ = 6.878, *p* < 0.001, and *d* = 1.300] misattributions in the High proficient group [Int: *t*_(27)_ = 1.570, *p* = 0.128, and *d* = 0.297; High: *t*_(27)_ = 3.498, *p* = 0.002, and *d* = 0.661].

To sum up, exploratory analyses on misinformation and source monitoring within the groups of participants with Intermediate and High levels of proficiency, overall, followed the patterns revealed by the main analyses. Regarding misinformation, results, on the one hand, confirmed the misinformation effect in both the groups and no difference in the degree of the effect depending on the language of misinformation presentation within the groups. On the other hand, between groups analysis revealed higher misinformation acceptance coming from the English narrative for more proficient participants. As for source monitoring, correct source attributions were significantly higher than the incorrect source attributions in all the conditions; however, there was no significant difference between the Russian and English sources. Analyses for incorrect attributions showed no significant differences between the Russian and None sources; however, the English-to-Russian source misattributions were significantly higher than the English-to-None misattributions only in the High proficient group. Confidence ratings complemented these patterns and for the most part, showed similar results as in the main analysis on confidence.

## Discussion

This experiment aimed to examine the possible effects of language on memory errors. To investigate this relationship, we used the misinformation paradigm and explicit source monitoring task. Our main expectations were that the participants would be better, or, worse at rejecting misleading information that was presented in their second language due to increased, or, decreased cognitive processing of this information. Analyses showed that the misinformation effect was present; however, there was no difference in the degree of misinformation acceptance between the Russian and English narratives. Other studies on the misinformation effect in bilinguals did not either report significant differences between the conditions (Shaw et al., 1997) or attributed it to language proficiency (Calvillo and Mills, 2020). Specifically, Calvillo and Mills (2020) reported an increased misinformation effect in less proficient language. This study had some limitations; however, as the sample was not properly balanced between the English-Spanish and Spanish-English bilinguals, and what is more important, the measure of language proficiency was performed in the form of object-naming task and self-assessment. Although self-assessment of the abilities of the participants can be useful, it can be argued that object-naming tasks could not be an entirely reliable measure of proficiency when participants have to process information in more complex structures, such as texts. Therefore, a more reliable measure is necessary to ensure that the observed effect is not caused by a lack of linguistic command. In the current experiment, we used objective measures as the main measure of second language proficiency. All the participants completed a Cambridge test, and analyses were based on mainly high-proficient (intermediate and higher levels of proficiency) participants. Thus, the absence of the expected interaction between misinformation endorsement and the language can be explained in that as people get more proficient in a second language, their interaction with information does not differ in both languages.

This conclusion was further supported by the exploratory analysis of misinformation effect and source monitoring between the participants with the Intermediate level of English language proficiency and participants with the higher levels of proficiency. Comparison of the degree of misinformation acceptance in English showed significant differences suggesting that the misinformation effect was greater for the High-proficient group than for the Intermediate group. A recent review of false memories in the bilingual Deese-Roediger-McDermott (DRM)-paradigm by Suarez and Beato (2021), also found that false memories are greater in participants with higher proficiency compared to the ones with lower levels and do not differ when the command of the participants in both the languages is similar. Importantly, the authors propose that it is not proficiency *per se* but rather dominance and environmental and interactional context that plays a major role in such differences (Beatty-Martínez et al., 2020; Suarez and Beato, 2021).

Indeed, both the results by Calvillo and Mills (2020), as well as the results of the current experiment could also be explained by the expectations, the participants have in a particular experimental setting and linguistic environment (Marian and Fausey, 2006), as well as their general higher confidence in their more proficient language. Representative to this notion is a study by Marian and Neisser (2000) examining a cued recall for autobiographical memories in Russian-English bilinguals. The results indicated that regardless of the language in which the cue was presented (Russian or English), the recall of the participants was mainly influenced by the linguistic environment of the experiment, i.e., when interactions between the experimenter and participant were overtly in the Russian language, recalled were the memories that had been mainly encoded in the Russian language context. Indeed, almost all of the materials (with the exception of the English narrative) and instructions and recognition tasks were presented in Russian, and therefore, the retrieval of information was conducted in Russian. The effect of the linguistic environment and the language of information retrieval could be further tested in the future using a mirroring experiment, in which English is the main language of the experiment, with some of the materials being in Russian, as well as the cross-linguistic paradigm introduced by Shaw et al. (1997).

In relation to source monitoring performance, our expectations of the differences in misinformation effect among the languages were not confirmed. However, the ANOVA revealed that participants favored Russian sources when making incorrect source attribution for the English source (i.e., when the original source of information was the English narrative). Analysis of confidence ratings complemented these results, showing higher confidence ratings in such misattributions. This implies that participants, indeed, treated this information as coming from a Russian source. There was no similar pattern for other observed sources (Russian and None). These results may be an indication that the participants might have invested more resources in processing the information in the English language, which was one of the theories underlying the hypotheses in the current research. As mentioned in the previous sections, the source monitoring framework argues that memory judgments are thought to be based on phenomenological cues and meaningful details that are assigned to particular sources (Raye and Johnson, 1980). In the case that information processing in English was more effortful, more cognitive information would be assigned to this source, making it more distinguishable in that dimension. Therefore, participants were able to recognize the information as coming from the narrative, but for some reason attributed it to the wrong source.

Alternatively, it could also be argued that observed differences in source misattributions were caused by the structural differences between the languages themselves. Although Russian and English do not have such specific grammar features like Japanese or Turkish, they still demonstrate many differences (absence or presence of case system, personal ending in verbs, declination of adjectives by gender, etc.) that can influence the source monitoring processes. So, in the future, the effect of these linguistic features on source monitoring could be investigated with more precision.

Finally, there might be one more possible explanation for this effect. As discussed, source confusion is considered to underlie the misinformation effect, resulting in individuals confusing the source of original information and the source of postevent information. On the one hand, the presence of the misinformation effect in the current experiment implies that participants confused the sources of original (video) and postevent (narratives) information. On the other hand, the results of source monitoring showing a preference for Russian narrative in the English source suggest that participants recognized that information came from the narrative but could not indicate the exact narrative. It was argued that it is more difficult to determine the origin of memories derived from external sources than, for example, to discriminate between the external and internal sources (Raye and Johnson, 1980). Memories from external sources are to be distinguished based on specific sensory content. Thus, it is more difficult to distinguish between the external sources when they are of the same or similar modality as sensory information related to these sources is similar.

In the case of our experiment, all the sources were external, but they differed in modality and could have provided different cues to justify source judgments. It might have been easier to distinguish between the information that came from the video and narratives (different modalities) than to discriminate between the information coming from two narratives (same modality). Therefore, participants were better at recognizing the modality of information, but not the source (English or Russian). In the future, the influence of modality can be further tested in other source monitoring paradigm, such as reality monitoring.

To our knowledge, this study is the first bilingual misinformation paradigm that did not manipulate the language of encoding and retrieval, but rather presented misleading information in two languages and measured its acceptance directly using explicit source monitoring tasks. The degree of misinformation acceptance did not seem to be affected by the language of misleading information; however, there might be still an influence on general information processing as shown by the results from the source monitoring task. Specifically, findings of the source monitoring task raise the basis for further examination of how exactly the monitoring processes work in bilinguals which can have important implications, both theoretically and practically.

## Data Availability Statement

The raw data supporting the conclusions of this article will be made available by the authors, without undue reservation.

## Ethics Statement

The studies involving human participants were reviewed and approved by the HSE University Ethics Committee. The patients/participants provided their written informed consent to participate in this study.

## Author Contributions

The conceptualization of the research study was conducted by AD and BM-L. Data collection was conducted by AD, and analyses by AD and BM-L. The initial writing was completed by AD. Both the authors revised and contributed to the final version.

## Funding

This article is an output of a research project implemented as a part of the Basic Research Program at the National Research University Higher School of Economics (HSE University).

## Conflict of Interest

The authors declare that the research was conducted in the absence of any commercial or financial relationships that could be construed as a potential conflict of interest.

## Publisher's Note

All claims expressed in this article are solely those of the authors and do not necessarily represent those of their affiliated organizations, or those of the publisher, the editors and the reviewers. Any product that may be evaluated in this article, or claim that may be made by its manufacturer, is not guaranteed or endorsed by the publisher.
