# Supplementary material for: False Memories in Native and Foreign Languages
Source: Front Psychol. 2021 Sep 28;12:716336. doi: 10.3389/fpsyg.2021.716336 (PMC8505984; doi:10.3389/fpsyg.2021.716336)
Supplement: Supplementary file 1 [file Data_Sheet_1.docx]

Supplementary Material

# Appendix A. Materials used in the experiment.

**Table 1.** True details extracted from the video

| № | **In Russian** | **In English**  **(not used in the experiment)** |
| --- | --- | --- |
| 1 | Кусты у дороги были густыми, сквозь них ничего не было видно. | The bushes along the road were dense, and nothing could be seen through them. |
| 2 | Машины были припаркованы на склоне дороги. | The road where the cars were parked had a pronounced slope |
| 3 | На руке молодого мужчины были часы. | The young man wore a watch. |
| 4 | Когда молодой мужчина вышел из машины, он посмотрел на машину, в которой находилась камера. | When the young man got out of the car, he looked at the car with the camera in it. |
| 5 | Второй мужчина был темноволосым и невысокого роста. | The second man was dark-haired and short. |
| 6 | Водитель не выходил из машины. | The driver did not leave the car. |
| 7 | Номерной знак машины, из которой вышли мужчины, содержал цифры "042". | The license plate of the car the men got out of contained the numbers "042". |
| 8 | Здание, видневшееся вдалеке, было красного цвета. | The building in the distance was red. |
| 9 | Отъезжая, машина начала движение до того, как двери закрылись. | Driving away, the car started moving before the doors were closed. |
| 10 | Молодой мужчина не закрыл дверь, когда вышел из машины. | The young man did not close the door when he got out of the car. |
| 11 | Машина, из которой молодой мужчина забрал вещи, стояла через одну от машины, в которой находилась камера. | The car from which the young man took things was parked one card down from the car in which the camera was located. |

**Table 2.** False details manipulated in the misinformation paradigm

| № | **In Russian** | **In English (not used in the recognition)** |
| --- | --- | --- |
| 1 | В небе были видны самолеты. | There were airplanes in the sky. |
| 2 | Одна из припаркованных машин была зеленого цвета. | One of the parked cars was green. |
| 3 | Молодой мужчина был низким и довольно толстым. | The young man was rather short and a little bit fat. |
| 4 | Второй мужчина курил. | The second man smoked. |
| 5 | Молодой мужчина нес черную спортивную сумку. | The young man carried a dark sports bag. |
| 6 | Невдалеке виднелся светофор. | There was a traffic light seen not far away. |
| 7 | Оба мужчины покинули машину через задние двери. | Both men left the car through the back doors. |
| 8 | На втором мужчине была футболка белого цвета. | The second man wore a white T-shirt. |
| 9 | Молодой мужчина сразу направился к припаркованной на обочине машине. | The young man went straight to the car parked on the said of the road. |
| 10 | Молодой мужчина нес вещи в левой руке. | The young man carried the bags in his left hand. |
| 11 | Дорогу окружали пустые поля. | There were empty fields on both sides of the road. |
| 12 | На молодом мужчине была черная футболка. | The young man wore a black T-shirt. |
| 13 | У молодого мужчины была отмычка. | The young man had a wrench. |
| 14 | У молодого мужчины была перчатка белого цвета. | The young man wore a white glove. |
| 15 | Перед тем как залезть в машину, молодой мужчина почесал шею. | Before entering the car, the young man scratched his neck |
| 16 | Машина, из которой вышли мужчины, была синего цвета. | The car the men left was blue. |
| 17 | Молодой мужчина вышел из машины через переднюю дверь. | The young man left the car through the front door. |
| 18 | Второй мужчина разговаривал с водителем. | The second man talked to the driver. |
| 19 | Молодой мужчина передал вещи второму мужчине перед тем, как они уехали. | The young man gave the bags to the second man before they left. |
| 20 | На другой стороне дороги была собака. | There was a dog on the other side of the road. |

## Narratives describing the crime event in the misinformation paradigm

## Narrative 1

***In English:***

My Russian friend Ivan and I went to the airport to pick up my mom, who was coming to visit me for the first time. We came to the airport early and discovered that her flight was delayed, so we decided to park at the side of the road nearby the airport; parking fees are expensive. From where we were, we could see *airplanes taking off and landing (1)*. There was a *car right in front of us, it was old and green (2).* We woke up early that morning and decided to have a nap while waiting for my mom’s plane. We did not have glasses, so we covered our heads with the blankets to avoid direct light. The blankets were very light though, so we still saw through them. After a while a car double parked, just in between our car and the next. A young man left the car*.* *He was rather short and a little bit fat (3)*. He was wearing a white short-sleeve T-shirt, black training trousers with white stripes on the sides, and dark flip-flops. There was also a second man. *This second man stayed by their car smoking (4)*. Suddenly, Ivan told me to remain calm and stay covered under the blanket. The first man went to the bushes and then reappeared hiding something behind his back. He down the string of cars. After less than 10 seconds, he went back to their car with something that looked like *a dark sports bag (5)*. He was wearing gloves. When both men entered the car, it drove away. I didn’t see any other people around. There were some buildings, but they were pretty far away. Shortly after the car left, Ivan put off his blanket and we discussed what we saw. We didn’t know how to characterize this, but we thought the men looked a little bit suspicious. Anyways, it was almost time to pick up my mom, so we left.

***In Russian:***

Мы с Джоном, моим другом из Америки, поехали в аэропорт встречать его маму, которое приезжала в гости. Ее самолет задержали, так что мы решили подождать ее на обочине шоссе, потому что на парковке выходило очень дорого. С места, где мы остановились, *было видно, как самолеты взлетают и заходят на посадку (1)*. Прямо перед нами стояла *старая зеленая машина (2).* В тот день мы проснулись очень рано, Джона клонило в сон, так что мы решили вздремнуть. У меня на заднем сидении было несколько легких полупрозрачных пледов, мы накрылись ими, чтобы солнце не светило в глаза. Где-то через час перед нами остановилась машина. Из нее вышел молодой мужчина. *Он был невысоким и довольно толстым (3).* На нем была белая футболка, темные спортивные штаны с полосками по бокам и темные шлепанцы. Потом вышел еще один мужчина. Ни один из них не обратил на нас внимания, потому что мы были под пледами. В это время молодой мужчина зашел в кусты на обочине, потом вышел оттуда, пряча что-то за спиной, и направился к одной из машин, припаркованных перед нами. Я понял, что происходит что-то неладное, и сказал Джону, чтобы он не высовывался из одеяла. *Второй мужчина остался у машины и закурил (4)*. Через какое-то время, довольно быстро, молодой мужчина вернулся *с черной спортивной сумкой (5).* На руках у него были перчатки. Потом оба мужчины залезли в машину, и они уехали. Какое-то время после этого мы еще посидели укрытые одеялами, потом сняли их. Нас обоих взволновало то, что мы увидели. Ситуация была подозрительной. Я не видел вокруг других людей, но заметил собаку на другой стороне дороги, возможно, ее хозяин был где-то неподалеку. Мы посидели еще какое-то время, обсуждая происшедшее, но, посмотрев на время, увидели, что самолет мамы Джона скоро приземлится, так что мы поехали в аэропорт.

## Narrative 2

***In English:***

My Russian friend Ivan and I went to the airport to pick up my mom, who was coming to visit me for the first time. We came to the airport early and discovered that her flight was delayed, so we decided to park at the side of the road nearby the airport; parking fees are expensive*. On both sides of the road where we stood, there were empty fields (1).* We woke up early that morning and decided to have a nap while waiting for my mom’s plane. We did not have glasses, so we covered our heads with the blankets to avoid direct light. The blankets were very light though, so we still saw through them. After a while, a car double parked, just in between our car and the next. A young man left the car. *He was wearing a black T-shirt, dark shorts with white stripes on the sides and white flip-flops (2).* There was also a second man, he stayed by the car, watching the cars passing by. Suddenly, Ivan told me to remain calm and stay covered under the blanket. The first man went to the bushes and *then reappeared hiding a wrench behind his back (3).* He down the string of cars. After less than 10 seconds, he went back to their car with something that looked like white plastic bags. *He was wearing a white glove (4).* *Before entering the car, he scratched his neck (5).* The second man also got in the car, and it drove away. I didn’t see any other people around. Shortly after the car left, Ivan put off his blanket and we discussed what we saw. We didn’t know how to characterize this, but we thought the men looked a little bit suspicious. Anyways, it was almost time to pick up my mom, so we left.

***In Russian:***

Мы с Джоном, моим другом из Америки, поехали в аэропорт встречать его маму, которое приезжала в гости. Ее самолет задержали, так что мы решили подождать ее на обочине шоссе, потому что на парковке выходило очень дорого. *С обеих сторон дороги были пустые поля (1)*. В тот день мы проснулись очень рано, Джона клонило в сон, так что мы решили вздремнуть. У меня на заднем сидении было несколько легких полупрозрачных пледов, мы накрылись ими, чтобы солнце не светило в глаза. Где-то через час перед нами остановилась машина. Из нее вышел молодой мужчина. *На нем была черная футболка, темные шорты с полосками по бокам и белые шлепанцы (2).* Потом вышел еще один мужчина. Ни один из них не обратил на нас внимания, потому что мы были под пледами. В это время молодой мужчина зашел в кусты на обочине, потом вышел оттуда, *пряча за спиной отмычку (3)*, и направился к одной из машин, припаркованных перед нами. Я понял, что происходит что-то неладное, и сказал Джону, чтобы он не высовывался из одеяла. Второй мужчина остался у машины, на которой они приехали, и смотрел по сторонам. Через какое-то время, довольно быстро, молодой мужчина вернулся с двумя белыми пакетами. На руке, в которой он нес пакеты, *была белая перчатка (4). Перед тем как залезть в машину, он почесал шею (5).* Второй мужчина тоже забрался в машину, и они уехали. Какое-то время после этого мы еще посидели укрытые одеялами, потом сняли их. Нас обоих взволновало то, что мы увидели. Ситуация была подозрительной. Я не видел вокруг других людей, но заметил собаку на другой стороне дороги, возможно, ее хозяин был где-то неподалеку. Мы посидели еще какое-то время, обсуждая происшедшее, но, посмотрев на время, увидели, что самолет мамы Джона скоро приземлится, так что мы поехали в аэропорт.

## Narrative 3

***In English:***

My Russian friend Ivan and I went to the airport to pick up my mom, who was coming to visit me for the first time. We came to the airport early and discovered that her flight was delayed, so we decided to park at the side of the road nearby the airport; parking fees are expensive. *We parked not far from the traffic light, and sometimes cars stopped on the red signal (1)*. We woke up early that morning and decided to have a nap while waiting for my mom’s plane. We did not have glasses, so we covered our heads with the blankets to avoid direct light. The blankets were very light though, so we still saw through them. After a while a car double parked, just in between our car and the next. At the same time *back doors opened and two men left the car (2)*. One was young. The second was older, *he was wearing a white short-sleeve T-shirt, black training trousers with white stripes on the sides, and dark flip-flops (3)*. Suddenly, Ivan told me to remain calm and stay covered under the blanket. *The first man went straight to one of the cars in front of ours (4).* It looked as if he was hiding something in his hand. The second man stayed by their car watching other cars pass by. After less than 10 seconds, this first man went back to their car with something that looked like two white plastic bags. *He was carrying them in his left hand (5).* While he was approaching the car, the second man entered the car for the front door. Before entering in the car, the young man threw the plastic bags to the backseat of the car. The car left after that. I didn’t see any other people around. Shortly after the car left, Ivan put off his blanket and we discussed what we saw. We didn’t know how to characterize this, but we thought the men looked a little bit suspicious. Anyways, it was almost time to pick up my mom, so we left.

***In Russian:***

Мы с Джоном, моим другом из Америки, поехали в аэропорт встречать его маму, которое приезжала в гости. Ее самолет задержали, так что мы решили подождать ее на обочине шоссе, потому что на парковке выходило очень дорого. *Мы остановились недалеко от светофора, и машины часто останавливались на красный (1).* В тот день мы проснулись очень рано, Джона клонило в сон, так что мы решили вздремнуть. У меня на заднем сидении было несколько легких полупрозрачных пледов, мы накрылись ими, чтобы солнце не светило в глаза. Где-то через час перед нами остановилась машина. *Задние двери открылись, и из машины вышли двое мужчин (2)*. Один был молодой. Второй выглядел старше, на *нем была белая футболка, черные спортивные штаны с белыми полосками и темные шлепанцы (3)*. Ни один из них не обратил на нас внимания, потому что мы были под пледами. *В это время молодой мужчина подошел к машине, стоявшей перед нашей (4)*. Я заметил, что он что-то прятал за спиной. Я понял, что происходит что-то неладное, и сказал Джону, чтобы он не высовывался из одеяла. Второй мужчина остался у машины, на которой они приехали, и смотрел по сторонам. Через какое-то время, довольно быстро, молодой мужчина вернулся, *в левой руке он нес два белых пакета (5).* На руках у него были перчатки. Увидев его, второй мужчина, который стоял до этого у машины, залез в машину и сел спереди. Молодой закинул пакеты на заднее сидение и тоже забрался в машину. После этого они уехали. Какое-то время после этого мы еще посидели укрытые одеялами, потом сняли их. Нас обоих взволновало то, что мы увидели. Ситуация была подозрительной. Я не видел вокруг других людей, но заметил собаку на другой стороне дороги, возможно, ее хозяин был где-то неподалеку. Мы посидели еще какое-то время, обсуждая происшедшее, но, посмотрев на время, увидели, что самолет мамы Джона скоро приземлится, так что мы поехали в аэропорт.

## Narrative 4

***In English:***

My Russian friend Ivan and I went to the airport to pick up my mom, who was coming to visit me for the first time. We came to the airport early and discovered that her flight was delayed, so we decided to park at the side of the road nearby the airport; parking fees are expensive. We woke up early that morning and decided to have a nap while waiting for my mom’s plane. We did not have glasses, so we covered our heads with the blankets to avoid direct light. The blankets were very light though, so we still saw through them. After a while *a blue car (1)* double parked, just in between our car and the next. *A young man left the car through the front door (2)*. There was also a second man, he stayed by the car, *talking to the driver (3)*. Suddenly, Ivan told me to remain calm and stay covered under the blanket. The young man went to the bushes and then reappeared hiding something behind his back. He then went down the string of the cars in front of us. After less than 10 seconds, he went back to their car with something that looked like two white plastic bags. *He gave them to the second man before entering the car (4).* The car left after that. I didn’t see any other people around, but *there was a dog walking on the other side of the road (5)*, its owner probably was not far away as well. Shortly after the car left, Ivan put off his blanket and we discussed what we saw. We didn’t know how to characterize this, but we thought the men looked a little bit suspicious. Anyways, it was almost time to pick up my mom, so we left.

***In Russian:***

Мы с Джоном, моим другом из Америки, поехали в аэропорт встречать его маму, которое приезжала в гости. Ее самолет задержали, так что мы решили подождать ее на обочине шоссе, потому что на парковке выходило очень дорого. В тот день мы проснулись очень рано, Джона клонило в сон, так что мы решили вздремнуть. У меня на заднем сидении было несколько легких полупрозрачных пледов, мы накрылись ими, чтобы солнце не светило в глаза. Где-то через час перед нами остановилась *синяя машина (1). Из передней двери вылез молодой человек (2).* На нем была белая футболка, темные спортивные штаны с полосками по бокам и темные шлепанцы. Потом вышел еще один мужчина. Ни один из них не обратил на нас внимания, потому что мы были под пледами. В это время молодой мужчина зашел в кусты на обочине, потом вышел оттуда, пряча что-то за спиной, и направился к одной из машин, припаркованных перед нами. Я понял, что происходит что-то неладное, и сказал Джону, чтобы он не высовывался из одеяла. Второй мужчина остался у машины, на которой они приехали, и *говорил о чем-то с водителем (3).* Через какое-то время, довольно быстро, молодой мужчина вернулся с двумя белыми пакетами. На руках у него были перчатки. Перед тем как залезть в машину, *он передал их второму мужчине (4).* После этого они уехали. Какое-то время после этого мы еще посидели укрытые одеялами, потом сняли их. Нас обоих взволновало то, что мы увидели. Ситуация была подозрительной. Я не видел вокруг других людей, *но заметил собаку на другой стороне дороги (5)*, возможно, ее хозяин был где-то неподалеку. Мы посидели еще какое-то время, обсуждая происшедшее, но, посмотрев на время, увидели, что самолет мамы Джона скоро приземлится, так что мы поехали в аэропорт.
